# Dangerous Liaisons? Psychiatry and Law in the Court of Protection—Expert Discourses of ‘Insight’ (and ‘Compliance’)

**DOI:** 10.1093/medlaw/fww027

**Published:** 2016-12-22

**Authors:** Paula Case

**Affiliations:** Senior Lecturer, School of Law and Social Justice, University of Liverpool, Liverpool, UK

**Keywords:** Capacity, Court of Protection, Insight, Psychiatry

## Abstract

A finding that ‘P’ (as the person who is subject to Court of Protection proceedings is known) lacks mental capacity is the trigger for exposing them to decision-making by others and the powers of the Court of Protection (CoP) which, in the words of Justice Hedley, can be ‘invasive and draconian’ (Hedley J in *PC v City of York Council* cited in [2013] EWCA Civ 478 [13]). Whilst the law asserts the upper hand in the assessment of mental capacity for persons who come before the CoP, it is the discipline of psychiatry, which dominates expert witness testimony in these proceedings. There are a number of implications of allowing psychiatry to dominate this terrain, not least that, as will be argued in this article, clinical discourse, which makes reference to non-statutory terminology such as ‘lack of insight’ and ‘non-compliance’ are imported into the business of capacity assessment. This terminology, if used lazily and without clear reference to the statutory criteria, has the potential to muddy the waters of assessing P’s capacity. At its worst, it can mask value judgements, which threaten to undermine the law’s ‘autonomy promoting’ provisions set out in the Mental Capacity Act 2005. Whilst it is not intended to discredit ‘insight’ as a concept in psychiatry, this article concludes that it has a proper context and that in the mental capacity context, decision-makers, lawyers, and advocates should exercise careful scrutiny of its use, and CoP judgments should carefully interrogate the language imported by expert witnesses.

## I. INTRODUCTION: MENTAL CAPACITY—PSYCHIATRY’S DOMINION?

The domain of mental capacity assessment defies categorisation—it is ‘… not straightforwardly medical, legal, biological or psychological’.^1^ In cases where P’s mental capacity is disputed, the Mental Capacity Act 2005 (MCA) applies a test that fuses clinical and legal competences. The statutory test for incapacity requires those alleging incapacity to demonstrate that P is ‘unable to make a decision’ because of an ‘impairment or disturbance in the functioning of the mind or brain’.^2^ In cases that come before the Court of Protection (CoP), the evidence of an expert witness (usually a psychiatrist) that P is suffering from an ‘impairment’ is generally accepted, but the courts claim to be the ultimate arbiter of whether P is in fact ‘unable to make a decision’.^3^

Although mental capacity is clearly not a purely clinical concept, it is indisputable that witnesses from the field of psychiatry far outnumber other expert witnesses in CoP hearings where mental capacity is in issue. This might, at least in part, be explained by the fact that cases coming before the CoP often concern a patient with a history of mental disorder, but it is also clear that the evidence of the psychiatrist is commissioned not only on whether P is affected by ‘impairment’, but is also routinely sought on whether P is ‘unable to make a decision’. Taking a sample of CoP judgments where P’s capacity is unpacked in detail, those judgments identify 53% of the expert witnesses as being psychiatrists, as compared with 20% being ‘other doctors’, 14% being social workers and 9% being psychologists.^4^

The number of consultant psychiatrists identified in judgments dealing with capacity, and the extent to which their competence is presumed to extend to assessing whether P is ‘unable to make a decision’, may give rise to a number of questions and concerns in and of themselves. The focus of this article, however, is on the frequent use of particular terms in evidence, imported from psychiatry, which do not feature in either the MCA or the accompanying Code of Practice. This article will first review the language of the statutory test for incapacity in the MCA, with an eye to unpacking the relevance of clinical judgement to capacity assessment (Section II), before narrowing the focus to usage of the terms ‘insight’ and ‘lack of insight’ in the context of assessing someone’s decision-making capacity. The provenance of ‘insight’ will then be explored, along with its uneasy relationship with the task of assessing mental capacity (Section III), before interrogating use of the concept by expert witnesses in a selection of judgments in the CoP (Section IV). It is argued here that the medical language of insight has at times been imported into legal argument and judgments regarding P’s mental capacity and that the density of this language can ‘obfuscate’ the social dimension of capacity, cloak paternalistic assumptions and disrupt the autonomy promoting provisions of the MCA, such as the presumption of capacity. It is therefore essential that practitioners and decision-makers are alert to the implications such language can have.

## II. THE MCA: UNPACKING THE RELEVANCE OF CLINICAL JUDGEMENT TO CAPACITY ASSESSMENT

Before exploring the discourses of expert witnesses in cases dealing with P’s mental capacity, this section seeks first to explain how the weight attached to clinical judgement about capacity is embedded in the statutory framework itself. The MCA laid down the statutory test for incapacity, the effect of which is to create a ‘four-pronged’ arrangement designed to protect P from premature findings of incapacity—these are: (i) the presumption of capacity in section 1(2); (ii) the fact that *incapacity* must be demonstrated by evidence of an ‘impairment’ satisfying the ‘diagnostic threshold’ in section 2(1) of the Act; (iii) there must be a failure to satisfy the ‘functional’ criteria for being able to decide (further elaborated in section 3(1)); and (iv) the requirement that the impairment must be *causing* the inability to decide.^5^

The language of this test provides a first port of call in unpacking the relevance of clinical expertise to the assessment of capacity. The provisions are described in the MCA Code of Practice as a two stage test, with the diagnostic threshold in section 2 constituting ‘stage 1’ and the functional test in section 3 being described as ‘stage 2’.^6^ Starting with ‘stage 1’, someone is defined as lacking capacity if they are ‘unable to make a decision’ on the issue at hand, that ‘inability’ being caused by an ‘impairment or disturbance of the mind or brain’. First, in requiring some identifiable ‘impairment or disturbance’ of mental function as a separate requirement from characteristics manifesting an inability to decide, the MCA introduced what is known as a ‘diagnostic threshold’ into capacity assessment. It will *usually* (but not always) be the case that before someone is found to lack capacity, they are diagnosed with a condition (whether mental disorder, learning disability, or brain damage).^7^

In terms of the epistemic role of psychiatry in capacity assessment, it is the diagnostic test which looms large. Clough regards the ordering of the MCA test as significant; in foregrounding ‘the existence of an impairment of or disturbance in the functioning of the mind or brain’, emphasis is placed on medical diagnostic criteria. This positioning, she seems to suggest, has encouraged a situation in which ‘judges often accept the professional’s view that the person lacks capacity without necessarily scrutinising the particular requirements outlined in the Act.’^8^ Given the inclusion and prominence afforded to the diagnostic test, it should probably come as no surprise that this test for incapacity would invite heavy reliance on medicine, specifically psychiatry, for capacity assessment.

‘Stage 2’ of the test for incapacity sets out the ‘functional’ characteristics of being ‘unable to decide’ as requiring the identified impairment or disturbance to be causing P to be either: unable to *understand* the information relevant to the decision; unable to *retain* the information; unable to use or weigh the information to arrive at a decision or unable to *communicate* the decision. An earlier common law version of the test had also required P to ‘believe’ the information relating to the decision.^9^ Some recognised the danger that the ‘belief’ requirement could conceal what was, in truth, a difference in values between P and the decision-maker. Dicta from post-MCA case law has suggested that the belief criterion lives on in the statutory test, for if someone does not ‘believe’ information relevant to the decision, they cannot be said to ‘understand’ it or be able to ‘use’ or ‘weigh’ it.^10^ This is arguably too strict an approach which risks pathologising those who refuse to accept treatment. Arguably P can have capacity if they can ‘understand’ that others think they are ill or that others think the risks of refusing treatment are too high, even if P does not ‘believe’ the other person’s view is correct.

When it comes to stage 2, the CoP has been eager to claim supreme authority. Speaking of the functional test, Munby J (as he then was) in *A Local Authority v A* stated: ‘only the court has the full picture. Experts are neither able nor expected to form an overview.’^11^ Yet in all of the fifty-seven judgments identified by the researcher where capacity is discussed in detail, it is clear that expert witnesses (just over half of whom are consultant psychiatrists) provide evidence not only on P’s ‘impairment’, but also on P’s ability to understand, retain, and use and weigh the information to reach a decision. In doing so, it is fairly common for expert witnesses to make reference to P’s ‘lack of insight’.

This article applies a sub-strand of medical law scholarship, which focuses on the use of ‘clinical euphemisms’ in legal judgments, to explore some of the implications that extensive reliance on psychiatry has for the law on mental capacity assessment.^12^ It argues, in particular, that experts’ liberal deployment of the concept of ‘insight’ if not kept within proper limits has the potential to corrupt the assessment of capacity in the CoP (and in practice), and can threaten to undermine the statutory presumption of capacity embodied in section 1(2) of the MCA where P is refusing care or is otherwise ‘uncooperative’.

## III. THE LANGUAGE OF PSYCHIATRY AND THE TROUBLE WITH ‘INSIGHT’

Although by no means present in every case, a significant number of ‘capacity’ judgments from the CoP include the evidence of expert witnesses referring to P as having ‘a lack of insight’. The clinical concept of ‘insight’ has its roots in the discipline of psychiatry but appears in neither the provisions of the MCA, nor the accompanying Code of Practice. Expressions of concern surrounding use of the terminology of ‘insight’ in decision-making governed by statute are not new.^13^ Groundbreaking empirical research in mental health review tribunals in both the UK and Australia identified use of the term by decision-makers as problematic. The main concern expressed in these studies was that, despite the appearance of a ‘shared’ meaning between physicians, usage of the term lacked transparency (not being defined in any relevant guidance). There has been no study of the use of the term ‘insight’ in evidence presented to the CoP. The following discussion demonstrates that the opacity of the ‘insight’ terminology is just the beginning of the problems which can be attributed to its use in the assessment of mental capacity under the MCA.^14^

### A. History of Insight in Psychiatry

A review of the function of ‘insight’ within psychiatry (and medicine generally) reveals that this ‘clinical centric’^15^ concept has a controversial history. ‘Absence of insight’, broadly meaning unawareness of the fact of being mentally ill, has been criticised as a ‘poorly understood phenomenon’^16^ with ‘poor construct validity, being differently defined in different studies’.^17^ Critics from within the medical profession have also suggested that ‘insight’ as used in psychiatry stands for professional imperialism and arrogance, ignoring cultural sensitivity and according ‘a pride of place to a single, rather narrow, perspective—that of scientific psychiatry’.^18^ In order to understand some of these criticisms, it may be helpful to examine the degree to which ‘insight’ has undergone extension in both form *and* function in psychiatry.

#### 1. Diagnostic Creep 

Absence of insight was originally regarded as a symptom of psychoses, but in particular was regarded as a hallmark symptom of schizophrenia.^19^ However, in what might be regarded as an example of ‘diagnostic creep’ the concept of lack of insight as a symptom of disorder has been extended to other conditions. It has been associated with anorexia nervosa^20^ and to some neurological disorders such as stroke, the dementias (eg Alzheimer’s disease)^21^, Huntington’s disease,^22^ and traumatic brain injuries. In these latter contexts, it is often referred to as ‘anosognosia’ (meaning an unawareness of some cognitive, sensory, or physical deficit where that unawareness is thought to have a biological cause).^23^ Clearly, whilst insight may have started out as a concept with significance for a single disorder, it is now of potential relevance to a great many people whose capacity is finally determined in the CoP.

#### 2. Increased Diversity in Aetiology of Insight

Medical literature reveals that as insight terminology became popularised within psychiatry, aetiological explanations for deficits of insight multiplied. In its early form, a lack of insight into illness tended to be explained as a psychological defence mechanism; an unconscious device of self-preservation. An ‘awareness deficit’ protected the patient’s psyche from the painful realisation of illness and operated as a barrier against stigma and impact on the individual’s self-identity. Modern research tends to validate this ‘psychological defence’ explanation^24^, suggesting that the development of insight into psychosis increases the risk of depression amongst patients and also of suicide ideation.^25^

Biological origins for insight deficit are, however, more commonly referenced in modern clinical texts. Research using neuropsychological tests has, for example, suggested organic causes for lack of insight in psychoses, namely damage to the frontal lobe or right parietal lobe, although there remains a lack of consensus as to exactly where the deficit is to be located.^26^ A number of other studies tend to agree that lack of insight in patients with schizophrenia is associated with more general cognitive deficit (patients with poor insight are commonly reported as performing poorly in, eg the ‘Wisconsin card sorting test’^27^). Such research tends to corroborate the organic explanation, as the results are generally reported as being consistent with the proposition that insight deficit is caused by lesions in specific sites of the brain or ‘lower grey matter volumes’. There also exists a great deal of recent research utilising imaging techniques, the results of which are also consistent with the idea that poor insight in such patients is associated with some form of frontal lobe/right hemisphere damage.^28^

There appears to be a broad consensus that organic causes lie behind a patient’s ‘lack of insight’, even if the evidence is not particularly strong^29^ and even if the exact locus for that explanation remains elusive. Nevertheless, the modern tendency to account for lack of insight by reference to some biological explanation tends to underscore the clinical authority given to assessments, which conclude that P ‘lacks insight’.^30^ Any behaviour associated with that lack of insight is thereby potentially pathologised.^31^ The result may underplay the possibilities that this person’s perceived lack of insight is in truth a playing down of risks in order to win an argument or a principled objection to the regime of care and treatment being proposed by the other party.

#### 3. The ‘Compliance Connection’

Aside from diagnostic creep and increased reference to the biological underpinnings of insight deficit, other changes in the definition of lack of insight have serious implications for its use in the assessment of mental capacity. Researchers frequently identify Aubrey Lewis as a key figure in developing the concept of insight in psychiatry. Lewis, writing about psychoses in the 1930s, defined insight as ‘a *correct* attitude to morbid change in oneself’^32^—an often cited definition that identifies insight as a metaphor for whether the patient accepts that they are ill. The emphasis on the ‘correctness’ of the patient’s attitude implies that disagreement with the psychiatrist on the fact of illness requires ‘correction’; a questionable assumption in the light of modern recognition that psychiatrists, like other medical professionals and lawyers sometimes get it wrong.^33^ Clearly Lewis’s definition speaks to a bygone era in which the process of diagnosis was assumed to be infallible and the patients’ perspective on their experiences was marginalised.

Professor Anthony David’s 1990 analysis is another common reference point for clinical research purporting to measure insight. David treats ‘insight’ as encompassing three overlapping constructs; (i) the patients’ understanding that they are mentally ill, (ii) their ability to ‘relabel’ mental events as pathological, and (iii) their compliance with a treatment regime.^34^ This third dimension has given rise to suggestions that for a patient to show insight they may need to demonstrate ‘compliance’ with the treatment being recommended.^35^ The ‘compliance connection’ intensifies the problematic nature of the concept of insight, the language of compliance being associated with an ideology of blaming the patient for conflict and deviant behaviour,^36^ and assuming and justifying medical authority.^37^ It is not hard to imagine how this compliance connection can amplify the risk that ‘lack of insight’ becomes a means by which conflict with treatment or care providers is explained as symptomatic of P’s disorder or lack of capacity or, in other words, that refusal or non-cooperation are ‘medicalised’. For example, in the context of schizophrenia, some researchers have suggested that levels of ‘non-compliance’ provide a good indicator of how ill someone is.^38^ The dangers of this conclusion, both in the context of treating schizophrenia and in extrapolating this conclusion to other conditions, are obvious.

This brief review of the provenance and evolution of the terminology of insight might be enough to convince us that caution should be exercised before importing the language of insight into capacity assessment. Yet it is undoubtedly the case that some of the expert witnesses appearing before the CoP regard P’s ‘lack of insight’ as a metaphor for incapacity.

## IV. THE RELATIONSHIP BETWEEN INSIGHT AND CAPACITY IN THE CoP: THREE JUDGMENTS

‘Insight’ was used as a search term in Bailii to trawl reported judgments from 2007 to 2015 where capacity was dealt with in detail in the CoP. The search revealed that of those health and welfare judgments where capacity was discussed in detail (57), around a third made at least passing reference to P’s ‘lack of insight’ and a good many of these referenced P’s ‘insight’ repeatedly. There were some instances of ‘insight’ being referred to in a strictly diagnostic sense (as confirming, for example, a particular diagnosis of Alzheimer’s/vascular dementia).^39^ In *Heart of England NHS Foundation Trust v JB*,^40^ Peter Jackson J referred to evidence that P lacked ‘insight’ by reason of not believing that she had a mental illness, namely, schizophrenia. When used in the former clear and restricted sense, the judge in the ‘capacity affirming’ judgment of *JB* had no difficulty in separating her lack of ‘insight’ from the issue of her ‘capacity’. The judgment in *JB* found that P lacked insight into her schizophrenia, but had capacity to decide on the issue of amputation. ^41^ *CC v STCC* provides another heartening example of judicial resistance to the notion that behaviour labelled as ‘lacking insight’ signals incapacity.^42^ Justice Baker discounted the suggestion that P’s extreme overuse of the lifeline service and promise not to persist if allowed to return home, demonstrated lack of insight and evidence of incapacity. Instead, P’s counsel argued (and the judge agreed) that she demonstrated ‘insight’ through her lucid description of the journey to court and awareness of the role of carers and detail of the care package she had been offered whilst at home.^43^

In a number of cases ‘insight’ is used by expert witnesses to address the issue of whether P meets the functional criteria of incapacity (i.e. whether P is not able to ‘understand’, ‘retain’ or ‘use and weigh’ the information to reach a decision for the purposes of section 3). Some of these cases (discussed below) highlight the risks associated with deploying insight terminology too liberally in capacity assessment. These risks might be regarded as, including:

- the potential for conclusions about ‘insight’ to trump the statutory criteria for capacity assessment;
- the failure to map conclusions about insight onto the statutory criteria, thereby obscuring transparency in capacity assessment, undermining P’s right to make unwise decisions protected by section 1(4) of the MCA; and
- potentially conflicting with the statutory principle in section 2(3)(b) that aspects of P’s behaviour should not be equated with incapacity.

Further, allowing lazy references to ‘insight’ increases the risk of ‘agreement’ being treated as a de facto requirement for capacity and the associated danger of medicalising P’s refusal or non-cooperation with the care or treatment being offered. The following interrogation of uses of ‘insight’ in expert evidence focuses on three cases: *PH v A Local Authority Wandsworth Clinical Commissioning Group V IA* and *London Borough of Islington v QR*, each one chosen because it highlights different means by which the undisciplined use of ‘insight’ can jeopardise the autonomy promoting provisions of the MCA.

### *A. The Pervasiveness of Insight Testimony and the ‘Minimisation of Problems’:* PH v A Local Authority

The use of insight in the 2011 case of *PH v A Local Authority*^44^ deserves particular comment, first of all because of its pervasiveness as a theme in the evidence. PH had been diagnosed with Huntingdon’s disease ten years previously and, shortly before this application, had been driven to ‘Y Court’, a residential home, by his partner under the pretence that his stay would be temporary. Before making this application, PH had telephoned the police a number of times asking to be ‘rescued’. In the course of PH’s challenge^45^ of the local authority’s ‘standard authorisation’ keeping him at the home, the court had to determine PH’s capacity to decide whether he should be accommodated at Y Court to receive treatment and care. Baker J found PH to lack capacity on this issue, preferring the evidence of the four experts who discussed PH’s insight over the evidence of the independent consultant psychiatrist (Dr Rickards) and PH’s partner. Whilst it is not argued that the outcome in PH should have been different, there are features of the evidence that are of particular relevance to this article.

The language of ‘insight’ permeates the evidence of all four of the expert witnesses who regarded PH as lacking capacity to decide where to live (a consultant psychiatrist, two General Practitioners, and a social worker). Their testimony referred repeatedly to ‘insight’ as a factor in their assessments, for example:

- ‘[PH has] … poor insight into his physical and mental health condition.’ (Dr C, General Practitioner)
- ‘… PH is very limited in insight about his care needs.’ (Dr A, consultant psychiatrist)
- ‘He lacks insight into the needs of other residents, not from malice but diminished comprehension.’ (Dr B, General Practitioner)
- ‘… due to PH's limited insight into his own abilities and care needs, he does not appear to be retaining information with regard to his place of residence or care needs … PH appeared to have no insight into the risks that would be present in the community.’ (D, social worker)

Although the problem of PH’s lack of insight is deployed repeatedly in expert evidence and in varying contexts, the judgment contains no signs of attempts to define what is meant by ‘insight’. These extracts illustrate that insight was being used not just as a reference to PH’s awareness of the extent of his decline, but in connection with his subjectively defined ‘care needs’ and in relation to external factors (the needs of others).

Another theme in *PH* was that this lack of insight into ‘needs’ was demonstrated in conversations with *PH*, by the fact that he ‘minimised’ problems identified by service providers, or could not ‘evaluate the practicalities’ of being cared for at home or had ‘no insight into the risks that would be present in the community’.^46^ It is worth noting that these assertions sound very like suggestions that *PH* did not ‘believe’ the information he was told about the risks of returning home. As with ‘belief’, equating insight with capacity brings us perilously close to equating capacity with agreement. An observation that P is ‘minimising the risks’ of not having treatment or of not returning home from a care setting can be interpreted in a variety of ways—it may indicate a cognitive deficit causing a failure to appreciate a risk which is obvious to all others, or it might be a strategic attempt by P to downplay aspects of a situation in order to maximise chances of the favoured outcome. This much seems suggested on the facts of *PH* itself in the evidence of the dissenting expert, Dr Rickards:

PH had not mentioned any disadvantages of going home or living anywhere else in the community and had been very reluctant to identify any advantages of staying at Y Court. Dr Rickards felt that he was so concerned about getting home that it was very difficult for him to speak of those factors…^47^

Evidence that P is ‘minimising the risks’ of a particular course of action or inaction in conversation does not tell us whether P’s decision-making faculties are defective or whether P is simply avoiding territory, which, if discussed, might be construed as a concession; a level of agreement with the other party’s point of view. In contrast, the judgment in *JB* (above) directly confronted the ambiguity of *JB*’s minimisation of risks and refused on the facts of the case to treat it as symptomatic of her disorder: *‘*Her tendency at times to be uncommunicative or avoidant and to minimise the risks of inaction are understandable human ways of dealing with her predicament and do not amount to incapacity.’^48^

The dangers of such heavy reliance by expert witnesses on the undefined notion of insight include that it can mask a conclusion that P lacks capacity because he disagrees with the professional view as to what is in his best interests. As far as the law is concerned, consideration of what is in P’s best interests as part of the process of assessing capacity is taboo.^49^ The blurring of capacity assessment with a judgment on what is in P’s best interests is a fundamental error, albeit one which clearly occurs in practice and has been referred to as the ‘concertina effect’.^50^ Coincidentally, the judgment from the Court of Appeal in *RB v Brighton & Hove City Council*^51^ cited the decision in *PH* with approval, saying that it provided a classic example of ‘a patient who, because of the impaired functioning of his brain, *did not understand his own best interests.*’^52^ These words offer an example of how easy it is to collapse the distinction between capacity and best interests.

### *B. Insight, Compliance,* *and the Problem of Conceptual Fusion:* Wandsworth Clinical Commissioning Group v IA

Evidence presented in the case of *Wandsworth Clinical Commissioning Group v IA*^53^ also demonstrates ‘insight’ again being deployed by four experts: a consultant psychiatrist, consultant psychologist *and* two consultant neuropsychiatrists. As a result, the narrative of the judgment demonstrates the potential for a finding of incapacity based on what I have called here ‘conceptual fusion’. *IA* was ready for discharge from hospital. His physical health was compromised by diabetes and his situation was complicated by a prior head injury. This injury had caused cognitive impairment which necessitated careful assessment of whether he had capacity to make decisions in relation to residence and care. Justice Cobb described the assessment of *IA*’s capacity as ‘particularly difficult and finely balanced’ but, disagreeing with the Official Solicitor, ultimately found that *IA did* have capacity to decide his medical treatment, residence and care and financial and property affairs. According to much of the expert evidence however, *IA* lacked insight into ‘his health problems’, ‘his cognitive and emotional problems’, but also external factors such as ‘the state of his housing’. Again, like *PH*, he was observed as having a tendency to minimise his problems.^54^ More concerning though was the interpretation of *IA*’s obstructive and uncooperative behaviour advanced by counsel on behalf of the Official Solicitor. The judge summarised this as suggesting that whilst *IA*:

‘appeared to’ understand and weigh up the information and to have reached a reasoned decision, … his subsequent ‘failure to consistently maintain that position’ demonstrated his lack of insight and the ‘deficit in his executive functioning’.^55^

This summary suggests an incredible claim that positive findings on the statutory criteria of ‘understanding’ and ‘weighing’ can be trumped by a lack of this elusive, *non-*statutory quality of ‘insight’. The ideas that (i) lack of insight may be symptomatic of certain disorders or impairment, and (ii) an insight deficit possibly predisposes P to refuse therapeutic recommendations may be valid propositions in themselves. But here the two ideas have become fused together in the evidence, so that a subsequent uncooperative attitude is suggested to be an indicator of malfunction in *IA*’s decision-making and therefore of incapacity—IA looks like an example of the ‘compliance connection’ in operation.

On this occasion the court refused to accede to the portrayal of uncooperative behaviour as part and parcel of P’s impairment. Instead, Justice Cobb construed *IA*’s ‘outbursts’ and ‘dogmatic pronouncements’ as attributable to his frustration at not being able to participate more in proceedings and at the slow progress being made rather than poor insight. In taking this view, he resisted the invitation to pathologise *IA*’s difficult behaviour and refused to medicalise his lack of cooperation.

### *C. The Need to Map Insight to the Functional Criteria:* London Borough of Islington v QR

Other interesting permutations are demonstrated in *QR,*^56^ a judgment which again comes dangerously close to treating refusal of the option on the table as flowing from the symptoms of *QR*’s mental health issues. It is a highly unusual case in which *QR* was regarded as having capacity in relation to nearly all aspects of her life, including litigation capacity^57^, but did not have capacity in relation to, *inter alia,* signing up (or not) to a supported living tenancy, which offered 24 h support and monitoring. *QR* had a diagnosis of schizophrenia and, under the terms of her Community Treatment Order (CTO), living independently was not an option. The CTO included a discretionary provision that QR could live at the ABC facility where she received treatment and care. *QR* did not wish to surrender her council flat as she hoped to live there again when independent living became an option in the future. She had been offered three supported living options but had not liked any of them and, if independent living was not possible, she had expressed a wish to stay in her current placement. Again, evidence on lack of insight appears to have played a substantial role in determining *QR*’s lack of capacity on these issues.

*QR*’s lack of insight is referenced by all three consultant psychiatrists (although one (Dr Akenzua) took the view that this lack of insight did not prevent her from being able to ‘weigh’). It was said that *QR* ‘lacked insight into her condition’ (schizophrenia), including the need to take medication. The judge concluded:

While she was aware that IAOT were proposing the move because it would provide a less restrictive environment for her, QR did not appear to attach weight to that aspect. She objected to 24 hour support as being regimented, like boarding school and denying her the privacy and independence that she seeks.^58^

The issue of *QR*’s insight seems to have muddied the waters in terms of applying the statutory test for incapacity. *QR*’s insight deficit, namely her lack of belief in the diagnosis of schizophrenia, was central to the decision that she lacked capacity. The court accepted the opinion of Dr Killaspy, consultant psychiatrist, who regarded insight into illness as central to *QR*’s capacity to make a decision. The judgment went on to adopt the eight items of ‘relevant information’ set out in Dr Killaspy’s evidence which P needed to understand in order to have capacity to sign a supported tenancy agreement.^59^ As *QR* did not understand that she was ‘… required to live in 24 h supported accommodation because of her mental illness and this is why she has to sign a supported tenancy agreement’,^60^ consequently, she could not understand the nature and *purpose* of that tenancy agreement (i.e. provision of 24-h support and oversight of medication).^61^ Yet this was a woman of considerable intelligence whose litigation capacity was not doubted and there was nothing in the judgment to suggest that she did not ‘understand’ that the purposes behind a supported living agreement were protection, supervision, and treatment. The fact that she was not in *agreement* with those purposes did not prevent her from ‘understanding’ them, or indeed from using or weighing them. It is arguable that all *QR* needed to ‘understand’ were the reasonably foreseeable consequences of refusing the supported tenancy, and, given that independent living was not an option, it is hard to see that the risk of not taking medication was engaged by refusing the tenancies being offered. The finding that *QR* did not have capacity to sign up/refuse to sign up to the tenancy because she did not believe she was ill in this case, comes very close to pathologising *QR*’s disagreement with the local authority’s view of her best interests.

Another of the unsettling features of the outcome in *QR* is the recognition in the evidence that even if she had shown ‘insight’ as to her schizophrenia and her need for medication, her decision would probably not have been any different. In fact *QR* had expressed rational reasons for wanting to stay in her current accommodation if independent living was not feasible:

She has a number of reservations about the supported living options which have been offered to her which would be shared by any woman: fear of being unsafe in a particular area, fear of being unsafe in relation to other tenants generally and in view of their mental health histories, anxiety when having to face a group of male workers. Dr K acknowledged that QR is sensitive to her vulnerability to sexual violence for good reason and that IAOT take that seriously. She did not like aspects of all 3 options such as room size, back yard. I acknowledge that all of these are good and rational reasons for being reluctant to move somewhere.^62^

There are other cases where there has been evidence that, if P’s impairment was removed from the equation, P would still have coherent reasons for their decision unrelated to any impairment or disturbance. In these cases, this fact seems to be treated as a good indicator that P has capacity.^63^ For example, in *Re SB*^64^, capacity to consent to a termination of pregnancy was established notwithstanding a diagnosis of bipolar disorder, a condition accompanied by some delusional beliefs about her husband and mother. The factor that ‘weighed most significantly’ with Justice Holman was that *SB* had expressed ‘a range of rational reasons’ for her decision to terminate her pregnancy, which were independent of any delusions.^65^ This distinction from the approach endorsed in *SB* underscores the fact that the importance attached to insight may have distorted the line of reasoning in the *QR* judgment. The *QR* case also provides an example of the pre-MCA criterion of ‘belief’ resurfacing and demonstrates its problematic nature.

## V. IMPORTING THE LANGUAGE OF INSIGHT: EXPLORING IMPLICATIONS FOR CAPACITY ASSESSMENT IN THE COP

How much does it matter if the language of insight and compliance is allowed to flourish in expert evidence on capacity assessment? There are a number of implications of the law endorsing the use of this language in the course of court judgments; some of these are problems at an operational, semantic level, whereas others exist at an ideological level.

### A. Insight and the Obscuring of Transparency in Capacity Assessment

At an operational level, references to the concept of insight compromise the transparency of capacity assessment. As Morris asserted in the sibling context of ‘best interests’ decision-making, it is vital that society can have faith in those assessments and that the criteria being utilised by practitioners and the judges are ‘transparent, consistent, and accepted as proper’.^66^ Where P stands to lose so much as a consequence of a determination that they lack capacity, the transparency of those decisions and the criteria by which they are reached is of vital importance. Lady Hale’s statement in *Dunhill v Burgin* provides a useful reminder of this: ‘people are assumed to have capacity to make their own decisions and should only be deprived of the right to do so *in clear cases*.’^67^ But the use of the undefined and ostensibly clinical terminology of insight stands in the way of the law’s need for transparency. In at least two of the cases discussed above where insight seemed to play a major role in constructing a view that P lacked capacity, the cases could perhaps have been classed as ‘borderline’. *QR* had litigation capacity (but apparently no capacity to decide whether to sign a supported living tenancy arrangement) and the judge in *IA*’s case described the determination of P’s capacity as ‘particularly difficult and finely balanced’.^68^ For insight to play such a prominent role in the expert evidence in these cases seems contrary to McCombe LJ’s warning in *PC v City of York Council* that where mental impairment has been judged as insufficient to rob P of capacity in all other fields, it is particularly important to delineate why and how that mental impairment can be causative of her inability to decide on this particular issue.^69^ Unless insight is clearly defined by the expert making use of the concept, that expert’s assessment does not meet the enhanced need for transparency in these finely balanced cases.

Some of the problems noted in *QR* are referable to this lack of transparency. The expert evidence fails to clearly map conclusions about insight onto the statutory criteria for incapacity. Does it matter if we are left unclear on whether insight is being used by the expert witness as a metaphor for P’s ‘understanding’ (under section 3(1)(a)) or the ability to ‘weigh’ the relevant information (under section 3(1)(c))? Arguably it does. Failure to map onto the statutory test means that the expert’s assessment remains opaque and more difficult to challenge. Also, if lack of insight is being used in a generic sense to mean a lack of understanding there is a problem here too. The connotations of the ordinary usage of the word insight is a ‘deep understanding’,^70^ which would seem to imply a more demanding standard than the law’s requirement that P should understand the rudimentary characteristics of the decision and the salient issues,^71^ and conflicts with judicial insistence that the threshold for capacity should not be set too high.^72^

### B. Insight and Pathologising Refusal

Scholarship provides compelling examples of legal judgments where events are redefined using clinical euphemisms. The effect of such language is often reported as being to remove or neutralise the moral character of the issues at stake.^73^ The substitution of explicitly ethical judgement with clinical terms brings with it certain hazards for, as Nettleton has argued, ‘medical opinion is increasingly attributed with ‘apparent facticity’, whereby value judgements are transformed into indisputable ‘facts’.^74^ It is argued here that medical language has at times been imported into legal argument and judgments regarding P’s mental capacity and that the density of this language can ‘obfuscate’ the social dimension of capacity, can cloak paternalistic assumptions and that practitioners and decision-makers need to be alert to the implications such language can have.

Bearing in mind the potential for value judgement to masquerade as medical fact, the deeper ideological problems of insight are rendered obvious if it is acknowledged that the assessment of P’s mental capacity in difficult cases, which reach the CoP is not an assessment which can or should be resolved by reference to clinical opinion alone. Foster and Miola have recently argued that in respect of key issues in medical law where the nature of the decision is ethical, ‘the law is not just entitled but is obliged to be the final arbiter.’^75^ Mental capacity assessment surely occupies one of these primarily ‘ethical’ domains in medical law. It is not ‘purely technical’; its place as a process which opens the door to ‘best interests’ decision-making by professionals means that the issue of P’s capacity will often comprise a value judgement^76^, informed by the decision-maker’s perspective on where the line between autonomy and best interests decisions should be drawn.

Just as the assessment of P’s best interests is not to be determined solely by reference to considerations of a medical nature^77^, it is right that the assessment of capacity is a matter of judgement, which should only be assisted *to a degree* by the application of clinical knowledge and expertise. Whilst statute provides the ‘intellectual framework’^78^ within which clinical assessments take place, expert witness testimony reveals that competing clinical terminology is often deployed. It is therefore crucial that when clinical language is brought to bear on the functional aspects of the legal test for incapacity, its use is subjected to careful scrutiny. Failure to do so increases the risk that a refusal of treatment or care, or uncooperative behaviour, are pathologised, triggering the assumption that P *must* lack capacity and robbing the statutory presumption of capacity in section 1(2) of the MCA of all meaning. Where P’s refusal or non-cooperation are pathologised through the medical device of insight, this can also be said to undermine P’s right to make unwise decisions (protected by section 1(4) of the MCA) and is at odds with the statutory principle that aspects of P’s behaviour should not be equated with incapacity (section 2(3)(b)). It also threatens to displace the ethos of the statutory framework which is to assess the *procedure* by which P is making decisions rather than the *content* of those decisions (section 1(4) and section 2(3)(b) taken together). Finally, it contravenes the law’s insistence that capacity determination must not be contaminated by the professional view of best interests.

The incentives in medicine and social care practice to favour approaches, which do have the effect of pathologising refusals are manifold. The primary objective of medicine is the maximisation of health, and therefore any route which minimises the perceived physical risks to the patient is bound to be preferred. Practising defensively in order to minimise the risk of liability and public recrimination will also point practitioners towards options that minimise physical risks. The law is clearly partly implicated in this cost benefit analysis, as damages for allowing a patient to come to physical harm clearly far outstrip levels payable (and therefore the symbolic force of such judgments) for infringement of autonomy.^79^ These are powerful incentives to take action to protect the physical health of the patient, even if that risks overriding patient autonomy. The law therefore poses a near impossible challenge—practitioners must respect autonomy, when nearly any risk assessment will tell them that paternalism works best. It is hardly surprising then that the literature on mental capacity assessment in health and welfare cases has long identified the tendency in some environments for capacity to only be assessed in cases of conflict and refusal.^80^ In 2013, the House of Lords Select Committee on the MCA heard evidence from a number of witnesses who had concerns that assessments of capacity in medical settings were only triggered when treatment was being refused.^81^ A 2013 study of mental capacity assessment in UK hospitals also raised the concern that most assessments were the product of a patient refusing treatment^82^ and research has suggested that persons refusing to accept a placement in a nursing or care home are often assumed to lack capacity by reason of their refusal alone.^83^ Further corroborative findings are to be found in *Homeward Bound* a 2012 study by Emmet and others of decisions to discharge patients from hospital. Although not explicitly critical of the use of the terminology of insight, the following comment is of particular resonance with the findings of this article: ‘Where assessors did not agree with patients’ decisions, they were prone to interpret the decision as lacking insight and, thus, the decision- maker as lacking capacity.’^84^ Taken together the findings set out above suggest that in a number of contexts, the fact of refusal in and of itself can too readily be treated by professionals dealing with the Act as giving rise to cause for concerns about capacity. Where this approach is adopted, refusals of care are in effect pathologised, a practice which is at odds with the philosophy underpinning the MCA of appraising people’s decision- making *processes* rather than the *content* of their decisions.^85^ This approach is also reminiscent of the defensive ‘flak jacket’ approach to obtaining consent,^86^ where the primary function of capacity assessment becomes one of protecting health care professionals from potential liability or reprisals, rather than supporting the autonomy and best interests of the patient. The use of the language of insight exacerbates the risk of perpetuating these practices which undermine the ethos of the MCA. It is hard to resist the conclusion that ‘insight’ (like the pre-MCA criterion of ‘belief’) provides a means for covertly sustaining clinical values, which are in conflict with those of the law.

## VI. CONCLUSION

This article has explored some of the implications of psychiatrists as the expert witness of choice in capacity assessment and the infiltration of medical terminology into capacity assessment in the CoP. The fact that capacity can be facilitated or enhanced by the form of communication used or by the presence or absence of support, speaks to its inherently ‘social’ essence^87^. This in itself provides strong justification for exploring with a critical eye the impact of psychiatry as a discipline, the influence of consultant psychiatrists as expert witnesses (and the language they bring with them) on the shape of this area of law. Psychiatry has an important role to play in a multidisciplinary generation of a better understanding of how to support and empower those who may have borderline capacity, but healthy scepticism is necessary, otherwise the relevance of the psychiatric view may be overstated and ‘mishandled’.^88^

The MCA specifies the criteria against which incapacity is to be assessed. Whilst these criteria clearly place a premium on clinical judgment via the ‘diagnostic’ threshold, the law asserts the upper hand in capacity assessment, particularly with respect to the functional criteria for incapacity in section 3. The application of these criteria is sometimes, however, obscured by the clinical terminology of insight. The fact that ‘insight’ has diagnostic significance lends it clinical authority, even when it is not being used in a diagnostic sense. This term, however, has a controversial history within its own discipline, and is a poor fit for the legal process where transparency is prized. Its use is often not clearly mapped onto the statutory criteria and it has the potential to cloak value judgements in the apparel of ‘clinical fact’. Uncritically accepting the use of ‘insight’ in expert evidence on capacity assessment without clear attempts to map this lack of insight onto the statutory test for incapacity gives credence to therapeutic values, which seem in conflict with many of the autonomy promoting provisions of the MCA.

The concept also appears to have been co-opted by other health and social care professionals, and there is a danger that an absence of insight is identified in cases where there is merely a failure to internalise the medical or professional viewpoint of P’s best interests. Where this occurs, the rejection of treatment/services can too easily be construed as a manifestation of a ‘lack of insight’ which in turn indicates disorder or incapacity. In short, a lack of cooperation is potentially pathologised. It is not the author’s intention to discredit ‘insight’ as a concept in psychiatry, but rather to suggest that it has a proper context, for example, where relevant to part of a diagnosis. Outside of that context, it should be used with extreme caution and with an appreciation of its problematic nature. Referring to patients as ‘lacking insight’ in the context of judging whether they have decision-making capacity has unfortunate connotations which are consistent with a medicalisation of capacity and risks subverting the presumption of capacity. It is therefore crucial that all players in the decision-making process guard against the non-specific use of ‘insight’ in capacity assessment. Equally important is that its use is interrogated by lawyers and the courts, if experts do not make it entirely clear how their observations about P’s insight map onto the statutory scheme for assessing P’s capacity.

## Footnotes

1 B Secker, ‘Labeling Patient (In)Competence: A Feminist Analysis of Medico-Legal Discourse’ (1999) 30(2) J Soc Phil 295, 297, and see P Jackson J, *Heart of England NHS Foundation Trust v JB* [2014] EWCOP 342, [38]: ‘Bearing in mind JB’s longstanding mental illness it is entirely appropriate that the core assessment of her capacity comes from psychiatrists, but other disciplines also have an important contribution to make.’

2 s 2(1).

3 *A Local Authority v A* [2010] EWCOP 1549, [66]; *CC v STCC* [2012] EWCOP 2136, [62] and *London Borough of Islington v QR* [2014] EWCOP 26, [84].

4 The remaining 4% comprised a matron, solicitors, a midwife, and a community health nurse. These figures were extracted from a sample of forty-five out of fifty-seven judgments where capacity was unpacked in detail. In twelve out of the fifty-seven judgments, it was not possible to clearly identify the background of the experts giving evidence. In the forty-five judgments where the experts’ background was clearly identified, a total of 118 expert witnesses gave evidence on P’s capacity—further details on file with the author.

5 Of course, these safeguards are in effect subverted by interim powers under s 48 of the MCA where the ‘gateway test for engagement of the court’s powers’ has been recognised as not requiring rebuttal of the presumption of capacity: ‘What is required, in my judgment, is simply sufficient evidence to justify a reasonable belief that P may lack ** **capacity in the relevant regard. There are various phrases which might be used to describe this, such as ‘good reason to believe’ or ‘serious cause for concern’ or ‘a real possibility’ that P lacks capacity, but the concept behind each of them is the same, and is really quite easily recognised’:** ***Re F* [2009] EWCOP B30.

6 *MCA Code of Practice* (2007). Most CoP judgments adopt the terms ‘stage 1’ and ‘stage 2’, despite the fact that the Code of Practice effectively reverses the order of the functional criteria and diagnostic threshold as set out in s 2(1). The Code is not hard law and the Court of Appeal judgment in *PC v City of York Council* [2013 ] EWCA Civ 478 warned that addressing the diagnostic threshold first could result in the causation requirement being overlooked. Nevertheless, CoP judgments have generally continued to follow the order suggested by the Code of Practice.

7 The Code of Practice at para 4.11 recognises that an impairment or disturbance may be identified without any disorder, examples including concussion and the symptoms of drug or alcohol use.

8 B Clough, ‘People Like That: Realising the Social Model in Mental Capacity Jurisprudence’ (2014) 23(1) Med LR 53, 56.

9 *Re C* [1994] 1 WLR 290.

10 *Local Authority X v MM* [2007] EWHC 2003,per Munby J, [81]. And for further discussion of the contentious ‘belief’ criterion, see N Allen, ‘Is Capacity Insight?’ (2009) J Mental Health *L* 165.

11 At (n 12) above. And again in *CC v STCC* [2012] EWCOP 2136, [84]: ‘I acknowledge that there is consensus amongst the professionals who gave evidence that KK has lost the capacity to make decisions concerning her residence. These opinions are of course important evidence, but as stated above it is the court alone that is in the position to weigh up all the evidence as to the functional test and thus it is the court that must make the ultimate decision.’ Finally in *London Borough of Islington v QR* [2014] EWCOP 26: ‘The decision on capacity is one for the judge to make’.

12 Some of the broader implications are explored by the author in ‘Negotiating the Domain of Mental Capacity: Clinical Judgement or Judicial Diagnosis?’ (forthcoming).

13 See, eg the excellent piece by Allen (n 10) 165.

14 The lack of definition has been regarded as problematic in other places—see, eg J Peay, *Tribunals on Trial* (Clarendon Press 1989) where ‘insight’ is mentioned as the first in a number of recurrent themes in mental health tribunal decision-making (see particularly 142–43). See also academics Diesfield and McKenna commenting on the prevalence of its use in mental health review tribunals in New Zealand, despite the absence of a definition. K Diesfield and B McKenna, *Insight and Other Puzzles: Undefined terms in the New Zealand Mental Health Review Tribunal* (Mental Health Commission 2005).

15 G Owen and others, ‘Mental Capacity and Decision Autonomy: an Interdisciplinary Challenge’ (2009) 51(1) Inquiry 79.

16 S Chaudhry and others, ‘Insight in Psychiatry: a Review’ (2014) 1(1) Univ Res J Med Sci 22.

17 A Beck-Sander, **‘**Is Insight into Psychosis Meaningful?’ (1998) J Mental Health 25, 31.

18 R Perkins and P Moodley, ‘The Arrogance of Insight’ (1993) 17 Psych Bull 233.

19 DSM-V.

20 R Arbel and others, ‘The Neurocognitive Basis of Insight into Illness in Anorexia Nervosa: a Pilot Metacognitive Study’ (2013) 209 Psych Res 604.

21 For its increasingly common use in the context of dementia, see J Bond and others, ‘Medicalization of Insight and Caregivers’ Responses to Risk in Dementia’ (2002) 1(3) *Dementia* 313.

22 A Ho, ‘Huntington's Disease Patients have Selective Problems with Insight’ (2006) 21(3) Mov Disord 385.

23 Chaudhry and others (n 19).

24 The ‘socio-cultural’ explanation offered by B Saravanan and others, ‘Culture and Insight Revisited’ (2004) 184 Brit J Psych*.* 107.

25 A Carroll and others, ‘Insight and Hopelessness in Forensic Patients with Schizophrenia’ (2004) 38(3) Australia and New Zealand J Psych 169.

26 M Orfei and others, ‘Unawareness of Illness in Neuropsychiatric Disorders: Phenomenological Certainty versus Etiopathogenic Vagueness’ (2008) 14(2) Neuroscientist 203.

27 M Cooke and others, ‘Disease, Deficit or Denial? Models of Poor Insight in PSYCHOSIS’ (2005) 112 Acta Psych Scand 4. Although there are studies which did not reach a positive correlation between lack of insight and performance in this test: S Rossell and others (2003) ‘Insight: Its Relationship with Cognitive Function, Brain Volume and Symptoms in Schizophrenia’ Psychological Medicine, 33, 111–19.

28 D Antonius and others, ‘White Matter Integrity and Lack of Insight in Schizophrenia and Schizoaffective Disorder’ (2011) 128 Schizophr Res  76; M Cooke and others, ‘Neurological Basis of Poor Insight in Psychosis: A Voxel-Based MRI Study’ (2008) 103 Schizophr Res 40; N Bedford and others, ‘Self-Evaluation in Schizophrenia: an fMRI Study with Implications for the Understanding of Insight’ (2012) 12 BMC Psych 106.

29 Cooke and others (n 30) suggesting that the paucity of evidence leans towards the conclusion that insight deficit is multifactorial.

30 Note that some of the expert witnesses refer to the organic ‘causes’ of insight deficit— eg in *GW v A Local Authority & Anor* [2014] EWCOP 20: ‘Dr Moore observed that difficulties with insight and awareness in HD are thought to be caused primarily by the degeneration of frontal-basal ganglia brain pathways and to a lesser extent the emotional impact of loss relating to the condition’ ([28]).

31 Biological explanations for behaviour tend to entrench medicalisation—P Conrad and J Schneider, *Medicalization of Deviance: From Badness to Sickness* (Temple University Press 1992).

32 A Lewis, ‘The Psychopathology of Insight’ (1934) British J Med Psychol 332 (emphasis added).

33 For example, consider the validity and reliability problems widely recognised as affecting psychiatric diagnoses and the celebrated study by D Rosenhan, ‘Being Sane in Insane Places’ (1973) 179 Science 250.

34 AS David, ‘Insight and Psychosis’ (1990) 156(6) Brit J Psych 798.

35 For other examples of connecting insight with compliance, see: X Amador, *Special Report - Poor Insight in Schizophrenia: Overview and Impact on Medication Compliance* (2003) <http://mentalillnesspolicy.org/medical/lack-of-insight-schizoprhenia.pdf> last accessed 7th August 2016; R Kemp and A David, **‘**Insight and Compliance’ in B Blackwell (ed), *Treatment Compliance and the Therapeutic Alliance* (Harwood 1997) 61. Note however that the Cooke (n 30) study tended to dispute any connection between ‘compliance’ in schizophrenia patients and grey matter volume.

36 J Donovan and D Blake ‘Patient Non-Compliance: Deviance or Reasoned Decision Making?’ (1992) 34 Soc Sci Med 507.

37 J Trostle, ‘Medical Compliance as Ideology’ (1988) 27 Soc Sci Med 1299.

38 See, eg in the specific context of schizophrenia, J McEvoy ‘The Relationship between Insight in Psychosis and Compliance with Medications’ in X Amador and A David (eds) *Insight and Psychosis* (OUP 2008). But cf research such as that by R McCabe and others, ‘Is There a Role for Compliance in the Assessment of Insight in Chronic Schizophrenia?’ (2000) 5(2) Psych Heal Med 173, which suggests only a weak connection between insight and compliance.

39 For example, *SCC v LM & Others* [2013] EWCOP 1137 (consistent with Alzheimer’s/vascular dementia).

40 [2014] EWCOP 342.

41 For a similar conclusion outside of the health and welfare context, see the fascinating judgment in: *Re P (capacity to tithe inheritance)* [2014] EWCOP B14.

42 [2012] EWCOP 2136.

43 See also *A Local Authority v TZ* [2013] EWCOP 2322 where the judge refers to TZ’s insight into sexual relations (seemingly as a substitute for ‘understanding’) as indicating capacity: ‘sometimes it's easy to make the right choice, sometimes it isn't, but I would try to make the right choice.’ This insight seemed to me to be well above the level of ‘rudimentary’ ability required.

44 [2011] EWCOP 1704.

45 Under s 21A of the MCA.

46 [2011] EWCOP 1704 [48].

47 [2011] EWCOP 1704 [26].

48 *Heart of England NHS Foundation Trust v JB* [2014] EWCOP 342 [39].

49 For example, the guidance issued by Dame Butler-Sloss (as she was then) in *Ms B v An NHS Trust* [2002] EWHC 429, [100]: ‘Considerations that the best interests of the patient would indicate that the decision should be to consent to treatment are irrelevant.’

50 V Williams and others, *Making Best Interest Decisions: People and Processes* (Mental Health Organisation 2012) 3.2.

51 [2014] EWCA Civ 561.

52 ibid [45] (emphasis added).

53 [2014] EWCOP 990.

54 [45] and [51].

55 [65].

56 [2014] EWCOP 26.

57 [115], applying the test in *Masterman-Lister v Brutton & Co* [2003] 1 WLR 1511.

58 [98].

59 [45].

60 ibid.

61 At [101]–[102].

62 At [106].

63 *Re C* [1994] 1 WLR 290—C’s reasons for refusing amputation included religious beliefs which placed importance on being buried ‘whole’, beliefs which were unrelated to his disorder. CF the case of *An NHS Trust v Ms T* [2004] EWHC 1279. T was found to lack capacity to refuse blood transfusions. If Ms T’s borderline personality disorder was removed from the equation, there was no evidence of Ms T having any coherent reason for refusing treatment (the reason for needing transfusion was the self-harm associated with her disorder and her reasons for refusal seemed based in part on her ‘misconception of reality’ that her blood was evil but also her wish to end the cycle of hospital admission that her disorder had caused).

64 *Re SB* [2013] EWCOP 1417.

65 [41].

66 AE Morris, ‘Selective Treatment of Irreversibly Impaired Infants: Decision-Making at the Threshold’ (2009) 17(3) Med L Rev 347.

67 [2014] UKSC 18 [2] (emphasis added).

68 [2014] EWCOP 990 [6].

69 [2013] EWCA Civ 478 [60].

70 Oxford English Dictionary.

71 Macur J in *LBJ v RYJ* [2010] EWHC 2665.

72 *Sheffield City Council v E & Anr* [2005] 2 WLR 953 *per* Munby J [144].

73 For example, G Sayers ‘Non-Voluntary Passive Euthanasia: The Social Consequences of Euphemisms’ (2007) 14 EJHL 221; A Fenwick, ‘Applying Best Interests to Persistent Vegetative State – a Principled Distortion?’ 24 (1998) JME 86, referencing the use of judicial semantics to support a delusion about the objectives and consequences of ‘ceasing treatment’. See also, eg Lord Goff’s judgment in *Airedale NHS Trust v Bland* [1993] AC 789, which can be argued to medicalise the decision by reducing the issue of best interests to the clinical issue of whether treatment is considered ‘futile’. On debate as to who decides ‘futility’, see H Brody, ‘The Physician’s Role in Determining Futility’ (1994) 42(8)  J Am Geriatr Soc 875; R Mohindra, ‘Medical Futility: A Conceptual Model’ (2007) 33(2) JME 71. R Forde, ‘Who is to Define the Futility of Treatment – the Patient or the Physician?’ (1998) 118 *Journal of the Norwegian Medical Association 2652* Cf the encouraging summary of Dr Danbury’s evidence in *An NHS Trust v DJ* [2012] EWCOP 3524, [55]–[56] and critical reading of the concept by Ward LJ in *Aintree University Hospitals Foundation NHS Trust v DJ* [2013] EWCA 65, [33]–[38].

74 S Nettleton, *Sociology of Health & Illness* (3rd edn, Polity Press 2013) 7.

75 C Foster and J Miola, ‘Who’s in Charge? The Relationship between Medical Law, Medical Ethics and Medical Morality?’ (2015) Med L 23(4) Med L R 505

76 Secker (n 1).

77 *Re S* [2001] Fam 15: ‘… it is for the court to decide what is in the best interests of S. The medical advice the court receives is, of course, of the greatest importance - but it is that, namely advice.' *(per* ButlerSloss LJ, 22).

78 M Kapp and D Mossman, ‘Measuring Decisional Capacity: Cautions on the Construction of a Capacimeter’ (1996) 2 Psych Pub Pol and Law 73.

79 For example, the conventional sum (then £15,000) for the infringement of autonomy in *Rees v Darlington Memorial Hospital NHS Trust* [2003] UKHL 52.

80 For example, T Grisso and P Appelbaum, *Assessing Competence to Consent to Treatment* (OUP 1998) 66: physicians were criticised for their willingness to overlook nearly any degree or impairment provided P agreed with their suggested treatment regime. See also Kapp and Mossman (n 81), ‘formal assessments almost invariably take place only when a patient refuses to accede to a medical recommendation.’

81 *Mental Capacity Act 2005: Post-legislative scrutiny* (House of Lords Select Committee on the Mental Capacity Act 2005 2013–14) 75.

82 I Sleeman and K Saunders, *‘*An Audit of Mental Capacity Assessment on General Medical Wards’ (2013) 8 Clin Eth 47.

83 Williams and others (n 53).

84 C Emmett and others, ‘Homeward Bound or Bound for a Home? Assessing the Capacity of Dementia Patients to Make Decisions about Hospital Discharge: Comparing Practice with Legal Standards’ (2013) Int J Law and Psych 72, 76.

85 s 1(4) MCA 2005.

86 From Lord Donaldson’s judgment in *Re W* [1993] Fam 64, 76.

87 For example, *V v R* [2011] EWHC 822—recognising that with family support P could have capacity to make her own decisions.

88 M Silberfeld and D Checkland, ‘Faulty Judgment, Expert Opinion and Decision Making Capacity’ (1999) 20 Theor Med Bioeth 377, 378.

